# Introduction to nanoclusters: from theory to application

**DOI:** 10.1039/d4na90062f

**Published:** 2024-06-10

**Authors:** Yi Gao, Daojian Cheng, Zhigang Wang

**Affiliations:** a Phonon Science Research Center for Carbon Dioxide, Shanghai Advanced Research Institute, Chinese Academy of Sciences Shanghai 201210 China gaoyi@sari.ac.cn; b State Key Laboratory of Organic-Inorganic Composites and College of Chemical Engineering, Beijing University of Chemical Technology Beijing 100029 China chengdj@mail.buct.edu.cn; c Key Laboratory of Material Simulation Methods & Software of Ministry of Education, College of Physics, Jilin University Changchun 130012 China wangzg@jlu.edu.cn; d Institute of Atomic and Molecular Physics, Jilin University Changchun 130012 China

## Abstract

Yi Gao, Daojian Cheng and Zhigang Wang introduce the *Nanoscale Advances* themed collection on Nanoclusters: from theory to application.
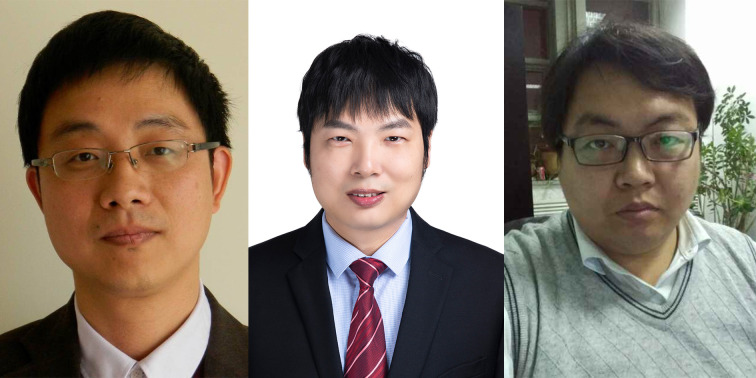

Since C_60_ was first successfully prepared in the 1980s, the vigour of nanoclusters has spurred wide applications in electronics, energy storage, catalysis, biosensors and nanomedicines, *etc.* Ultra-small size and unique structures are the key for their distinct properties. Conventionally, spectroscopic experiments, such as photoelectron spectroscopy, combined with the first-principles calculations have been proven to be a powerful tool for determining the structures in vacuum and the gas phase. Since the millennium, significant progress has been achieved in synthesizing clusters with specific morphologies and/or atomic structures in liquid, which prompts the preparation of nanoclusters in large amounts and tunes their properties to atomic precision. This kindles intense interest in the fundamental understanding of the evolution of the structures of nanoclusters and their corresponding structure–activity relationships.

On this specific theme, we present 16 articles, ranging from basic research, such as the study of atomic structures and the interaction between the clusters and other materials, to their precise synthesis, and further extend to the applications in a variety of frontier areas, including optics, biomedical imaging, the semiconductor industry, *etc.*

## Structures

Characterizing the structures of clusters lays the bedrock to understanding their properties and applications. Wang *et al.* combined the state-of-the-art spectroscopy techniques with quantum chemical calculations to reveal the structures and size effects of H_2_O–metal clusters (https://doi.org/10.1039/D3NA00873H). Ji *et al.* designed the metallocene nanowires in different shapes and predicted their magnetic and electronic characteristics (https://doi.org/10.1039/D3NA00926B). Cai *et al.* introduced a machine-learning method to construct the high-dimensional neural network to resolve the complexity of the configurational space of cerium oxide clusters (https://doi.org/10.1039/D3NA01119D). Han *et al.* applied the grand unified model to predict three medium-sized ligand-protected Au clusters and further confirmed their thermal/chemical stabilities and optical spectra by using density functional theory calculations (https://doi.org/10.1039/D3NA00372H).

## Interaction

Li *et al.* applied molecular dynamics simulations to show a gold surface could induce the conformational change of proteins (https://doi.org/10.1039/D3NA00185G). Fouegue *et al.* theoretically demonstrated the drug delivery potential of pure/doped C_24_ fullerene (https://doi.org/10.1039/D3NA00402C).

## Synthesis

Clusters synthesized with different structures exhibit different properties. Miliaieva *et al.* demonstrated the distinct electronic characteristics of nanodiamond clusters according to the different synthesis methods used (https://doi.org/10.1039/D3NA00205E). Schmitt *et al.* presented a novel method to the fully scalable, continuous flow synthesis of atom-precise Pt nanoclusters (https://doi.org/10.1039/D4NA00074A). Pluta *et al.* and Veedu *et al.* reported the methods to synthesize quantum dots presenting enhanced NIR photoluminescence (https://doi.org/10.1039/D3NA00404J, https://doi.org/10.1039/D3NA00869J). Vu *et al.* reported a facile way to synthesize alloys with improved surface-enhanced Raman scattering performance (https://doi.org/10.1039/D3NA00483J).

## Applications

The discrete energy levels of nanoclusters provide the versality to tune their optical, electronic, and chemical properties, which are important for their applications. Xia *et al.* took advantage of the chemo-photothermal effect by light-initiated aggregation of gold nanoparticles for tumor therapy (https://doi.org/10.1039/D3NA00114H). Super-resolution bioimaging was fulfilled by using the dual-color core–shell SiO_2_ nanoparticles (https://doi.org/10.1039/D3NA00310H). The promoted spin polarization with tunable magnetic properties of nanosized MXene were reported by Vénosová and Karlický (https://doi.org/10.1039/D3NA00474K). Tseng *et al.* unveiled the capability of Hf clusters to enable high resolution EUV photoresists (https://doi.org/10.1039/D3NA00508A). Copper hydride clusters were good catalysts for the hydrogenation and reduction reactions (https://doi.org/10.1039/D3NA01145C).

Nowadays, fast development of high-resolution characterization methods and artificial intelligence give new opportunities for characterizing the dynamic behaviors of clusters and designing new functional materials, which will witness a prospering era of cluster science and technology.

